# Acute Abdominal Pain and a Whirlpool Sign on Computed Tomography: A Case Report

**DOI:** 10.5811/cpcem.1394

**Published:** 2024-02-13

**Authors:** Christopher Libby, Evan Stern, Robyn Hoelle

**Affiliations:** * Cedars Sinai Health System, Department of Emergency Medicine, Los Angeles, California; † HCA Florida North Florida Hospital, Department of Graduate Medical Education, Gainesville, Florida; ‡ University of Central Florida/HCA Florida Healthcare, Gainesville, Florida

**Keywords:** mesenteric volvulus, malrotation, midgut volvulus, whirlpool sign, acute abdominal pain

## Abstract

**Introduction:**

Mesenteric volvulus is a rare cause of abdominal pain and bowel obstruction in elderly patients. When a mesenteric volvulus occurs in adult patients, the symptoms are often non-specific, which contributes to delays in diagnosis.

**Case Report:**

We present a case of a 75-year-old female who presented with non-specific abdominal pain. The rare whirlpool sign on computed tomography identified a mesenteric volvulus as the cause of small bowel obstruction. She was taken to the operating room and, after successful resection of the small bowel, she recovered and ultimately was discharged home.

**Conclusion:**

Early identification of a whirlpool sign and early surgical consultation are key to providing the best chance for salvage of ischemic small bowel due to mesenteric volvulus and to prevent a fatal outcome.

Population Health Research Capsule What do we already know about this clinical entity?

*Mesenteric volvulus is a rare cause of small bowel obstruction. It can be difficult to distinguish from other causes without appropriate imaging.*

What makes this presentation of disease reportable?

*The whirlpool sign— swirling of the mesenteric vessels on computed tomography (CT) of the abdomen—is a classic finding for a mesenteric volvulus.*

What is the major learning point?

*A mesenteric volvulus is a surgical emergency, and delays in diagnosis in emergency department patients increase morbidity and mortality.*

How might this improve emergency medicine practice?

*When identifying a whirlpool sign on abdominal CT, emergency physicians should consider midgut volvulus and obtain surgical consultation.*

## INTRODUCTION

While approximately 90% of midgut volvulus cases occur before the age of one year, cases are identified in patients of all ages.^1^ Adult-onset midgut volvulus is especially rare with an incidence of only 0.2–0.5%.^2^^,^^3^ When adults present with midgut volvulus, the condition presents as acute onset only 10–15% of the time.^4^ Acute onset midgut volvulus presents similarly to patients with acute bowel obstructions; however, subacute presentations present more insidiously. Patients with subacute to chronic presentations may have non-specific gastrointestinal symptoms such as cramping, bloating, weight loss, nausea, and vomiting that may come and go for weeks to months until an acute presentation or a diagnosis is made on advanced imaging. Without a high degree of suspicion, patients with subacute midgut volvulus may suffer from a delay in diagnosis in the emergency department (ED) and definitive surgical treatment.^5^ In the patient presented here, initial symptoms were suggestive of numerous abdominal pathologies. Diagnosis was ultimately revealed on computed tomography (CT) by identification of the whirlpool sign, which prompted life-saving surgical intervention.

## CASE REPORT

A 75-year-old woman presented to the ED for evaluation of sudden onset of left lower quadrant abdominal pain. She reported that she began feeling the abdominal pain approximately four hours prior to arrival but did not come to the ED until she began to experience nausea and dry heaving with associated chills. She reported passing flatus but had not had a bowel movement in the preceding 24 hours. She denied any recent trauma to the abdomen or pelvis and did not report any abdominal surgical history. The patient reported a vague history of “abdominal infections” without a diagnosis by her primary care physician or gastroenterology. She denied drinking well water, traveling recently, or camping. She reported a history of pulmonary emboli 10 years prior, but held her warfarin dose that morning because her international normalized ratio (INR) was “high.”

On presentation the patient was in no acute distress but was clammy and tachycardic at a rate of 118 beats per minute. On exam, her abdomen was diffusely tender without distention or peritoneal signs. She had an elevated white blood cell count at 17 × 10_3_ per microliter (K/μL) (reference range 4.5–11.0 K/μL), an INR of 5.5, and a lactic acid of 3.7 millimoles per liter (mmol/L) (0.4–2.0 mmol/L). An abdomen and pelvis CT with intravenous (IV) contrast showed findings suspicious for closed loop, small bowel obstruction. The CT also demonstrated evidence of swirling of the mesenteric root, known as a whirlpool sign (Image, Video). The findings of the whirlpool sign on CT suggested mesenteric volvulus as the cause of her bowel obstruction and prompted emergent surgical consultation.

**Image.**
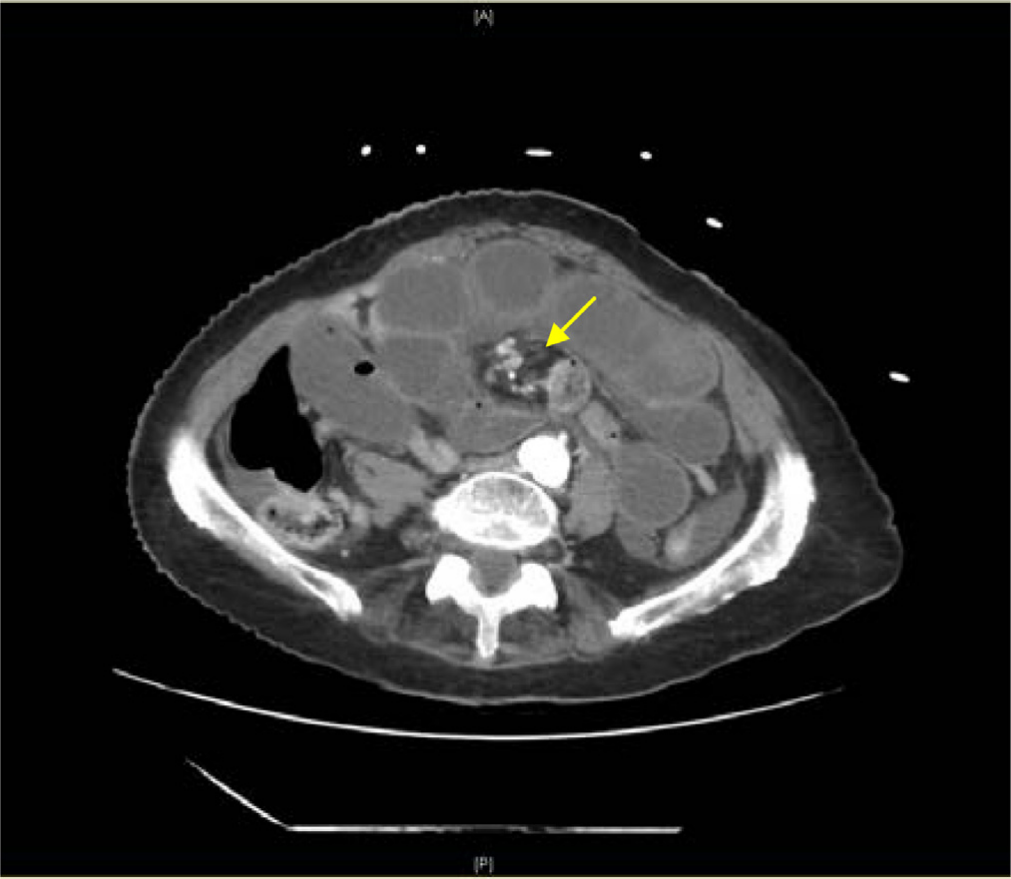
Computed tomography abdomen with intravenous contrast demonstrating dilated, fluid-filled loops of small bowel and a pattern of swirling mesenteric vessels called the whirlpool sign (arrow).

The patient received fresh frozen plasma and vitamin K for reversal of her INR in preparation for emergent surgery. Intraoperatively, her mesenteric volvulus was reduced and eight feet (∼243 cm) of jejunum was resected due to ischemia. She remained nil-per-os for seven days post-surgery before beginning a clear liquid diet, and she remained in the hospital for a total of nine days before being discharged to acute rehabilitation. She continued to recover and returned to baseline activities over the following month.

## DISCUSSION

Although history, physical exam, and laboratory tests aid in diagnosing midgut volvulus, imaging is the most useful. Abdominal radiographs are very quick and can be done in the ED at bedside to rule out other causes of abdominal pathology. However, radiographs are often inconclusive in midgut volvulus because even a positive “double bubble” sign indicating a small bowel obstruction does not rule out a concomitant midgut volvulus.^7^ Computed tomography with IV contrast gives much greater detail of the abdomen and can provide evidence suggestive of a midgut volvulus. Classic CT imaging findings include a whirlpool sign of twisted mesentery, malrotated bowel configuration, inverted superior mesenteric artery and superior mesenteric vein relationship, bowel obstruction, and free fluid/free gas in advanced cases.^8^ The whirlpool sign seen on CT represents the mesentery and superior mesenteric vein wrapping around the superior mesenteric artery in a counterclockwise direction.

The diagnosis of a midgut volvulus is considered a surgical emergency.^9^ Surgical consultation should not be delayed for additional testing once the history and imaging are suggestive of a volvulus. Time to surgical correction is the most important factor in mortality, which ranges from 0–25% in acute onset volvulus like our patient presented.^6^ When corrected before necrosis of the bowel has occurred, mortality can decrease to as low as 3–9%.^1^ In addition to standard treatment for a bowel obstruction, antibiotics covering against anaerobes and gram-negative organisms of the gut flora should be given to patients with midgut volvulus due to the high risk of translocation of bacteria secondary to bowel ischemia.^10^

## CONCLUSION

Abdominal pain in the elderly population is a common chief complaint in the ED with many etiologies. Small bowel obstructions with concomitant mesenteric volvulus is a surgical emergency that requires early identification and surgical consultation, which can often be missed in subacute presentations with non-specific gastrointestinal complaints. Diagnosis by CT abdomen and pelvis with contrast remains the imaging modality of choice to identify midgut volvulus. Emergency clinicians should be familiar with the common CT findings for both small bowel obstructions and the mesenteric whirlpool sign suggestive of midgut volvulus, as it drastically changes the patient’s prognosis and need for emergent surgical care.

## Supplementary Information

Video. Computed tomography of the abdomen and pelvis with intravenous contrast shows the superior mesenteric artery branching off from the aorta and ultimately swirling with the mesentery to create the whirlpool sign, diagnostic of a midgut volvulus. Additionally, the swirl serves as a transition point for the small bowel obstruction seen in the video as dilated loops of fluid-filled small bowel.
